# Heredity, education, developmental characteristics of children with somatoform disorders

**DOI:** 10.1192/j.eurpsy.2023.842

**Published:** 2023-07-19

**Authors:** M. Kalinina, G. Kozlovskaya, L. Baz, M. Ivanov

**Affiliations:** 1 child psychiatry; 2 сhild department, FSBSI MHRC; 3 Moscow Institute of Psychoanalysis, Moscow; 4 child psychiatry, FSBSI MHRC, Мoscow, Russian Federation

## Abstract

**Introduction:**

Psychosomatic disorders, their polymorphism and wide distribution in the population are the subject of study by many specialists in borderline mental disorders.

**Objectives:**

We examined 48 (19 boys, 29 girls) children aged 6-13 years who were referred for treatment to a pediatric hospital with suspected cardiac or respiratory pathology.

**Methods:**

Standard clinical methods (pediatric, psychopathologic, neurological, vegetative, psychological) were used. The mental state of children was assessed qualitatively, taking into account the data of psychopathologic, psychological examinations, as well as quantitatively, according to original questionnaires.

**Results:**

The clinical picture of the mental state was determined by neurotic disorders of the anxiety-suspecting hysterical type, and in 14% with transient psychotic episodes, qualified as an outpost, the symptoms of an endogenous disease.

Neurophysiologic tests revealed disturbances in the process of lateralization, visual perception and information processing with weakness of the right hemispheric, less often left hemisphere functions.

Neurological examination revealed some scattered symptoms of minimal cerebral dysfunction, as well as non-localized neurological signs in the area of cerebral innervation, there were signs of mixed vascular dystonia.

An analysis of environmental factors showed that children were brought up in conditions of insufficient attention, hypopedia. One third of the cases came from incomplete families. About a quarter of the children grew up in large families, were the youngest children of elderly parents.

In heredity, cases of manifest psychosis were not identified. However, an analysis of the personal qualities of parents speaks of schizotypical stigmatization; in almost every family, fathers or mothers had coronary heart disease and joint damage. Insufficient level of education of some parents.

**Conclusions:**

In general, the mental state of children, we can conclude that it corresponds to dysontogenetic with a predominance of schizotypical stigmas in half of them, partial underdevelopment of the sensory and emotional-volitional spheres, similar to disorders in children from conditions of maternal deprivation.

**Disclosure of Interest:**

None Declared

